# Amygdala subnuclei are differentially affected in the different genetic and pathological forms of frontotemporal dementia

**DOI:** 10.1016/j.dadm.2018.12.006

**Published:** 2019-01-25

**Authors:** Martina Bocchetta, Juan Eugenio Iglesias, David M. Cash, Jason D. Warren, Jonathan D. Rohrer

**Affiliations:** a Dementia Research Centre, Department of Neurodegenerative Disease, UCL Queen Square Institute of Neurology, University College London, London, United Kingdom; b Centre for Medical Image Computing, Department of Medical Physics and Biomedical Engineering, University College London, London, United Kingdom

**Keywords:** Amygdala, Frontotemporal dementia, Imaging, MRI, Volumetry

## Abstract

**Introduction:**

Frontotemporal dementia (FTD) is a heterogeneous neurodegenerative disorder with multiple genetic and pathological causes. It is characterized by both cortical and subcortical atrophies, with previous studies showing early involvement of the amygdala. However, no prior study has specifically investigated the atrophy of different subnuclei of the amygdala.

**Methods:**

Using an automated segmentation tool for T1-weighted volumetric magnetic resonance imaging, we investigated amygdalar subnuclei (AS) involvement in a cohort of 132 patients with genetic or pathologically confirmed FTD (age: mean = 61 years (standard deviation = 8); disease duration: 5 (3) years) compared with 107 age-matched controls.

**Results:**

AS were affected in all genetic and pathological forms of FTD. *MAPT* mutations/FTDP-17, Pick's disease, and transactive response DNA binding protein 43 kDa type C were the forms with the smallest amygdala (35%–50% smaller than controls in the most affected hemisphere, *P* < .0005). In most FTD groups, medial subnuclei (particularly the superficial, accessory basal and basal/paralaminar subnuclei) tended to be affected more than the lateral subnuclei, except for the progressive supranuclear palsy group, in which the corticoamygdaloid transition area was the least-affected area.

**Discussion:**

Differential involvement of the AS was seen in the different genetic and pathological forms of FTD. In general, the most affected subnuclei were the superficial, accessory basal and basal/paralaminar subnuclei, which form part of a network of regions that control reward and emotion regulation, functions known to be particularly affected in FTD.

## Highlights

• Amygdalar subnuclei are affected in all genetic and pathological forms of frontotemporal dementia.
• Amygdalar subnuclei are differently involved among genetic and pathological forms.
• *MAPT*/FTDP-17, Pick's disease, and TDP-43 type C showed the smallest amygdala.
• The medial subnuclei tended to be affected more than the lateral subnuclei.

## 1 Introduction

Frontotemporal dementia (FTD) is a heterogeneous neurodegenerative disorder. Genetically, around a third of patients with FTD have an autosomal dominant mutation in microtubule-associated protein tau (*MAPT*), progranulin (*GRN*), and chromosome 9 open reading frame 72 (*C9orf72*) [1]. Neuropathologically, the three most common abnormalities in the brain are tau, transactive response DNA binding protein 43 kDa (TDP-43), and fused in sarcoma inclusions [2], [3]. Atrophy in the medial temporal lobe is a common feature in cases of FTD, and the amygdala is often affected in the early stages of the illness, particularly in carriers of mutations in the *MAPT* gene [4], [5] where volume loss has been seen on magnetic resonance imaging 10-15 years before the expected onset [6]. Neuropathological investigations have also shown amygdalar involvement, e.g., one study described severe volume loss of 52% in FTD [7], where Pick's bodies were found in half of the sample, whereas another study showed TDP-43 inclusions in the basolateral nucleus of the amygdala in the earliest stage of the disease [8].

**Fig. 1:**
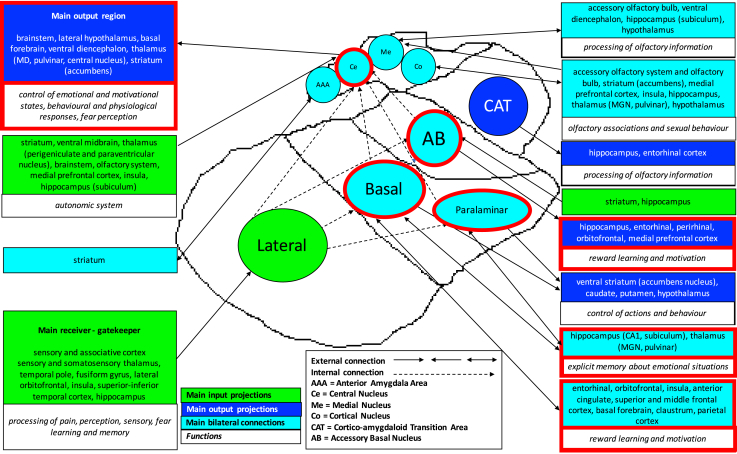
Schematic representation of the amygdala subnuclei and their functions and connections. Red-bordered boxes and subnuclei are part of the limbic system. The graph is based on the following studies [9], [10], [11], [13], [14]. Anatomical delineation of the subnuclei is based on the study by Saygin et al. [15].

The amygdala is composed of different subnuclei with connections to the limbic system, as well as to the rest of the brain [9], [10], [11], [12]. These are involved in reward learning, motivation, emotion, and several cognitive functions such as attention, perception, and explicit memory [10] (Fig. 1). Owing to recent advances in parcellation methods, it is possible to measure these amygdalar subnuclei (AS) *in vivo* on magnetic resonance scans [15]. We aimed to investigate the pattern of atrophy of the AS in a cohort with a genetic or pathologically confirmed diagnosis of FTD to clarify whether and to what extent the AS are impaired across these different forms of FTD.

## 2 Methods

We reviewed the UCL Dementia Research Center FTD magnetic resonance imaging (MRI) database to identify patients with a genetic or pathologically confirmed diagnosis of FTD and a usable T1-weighted magnetic resonance scan. A total of 132 patients were identified (Table 1). Seventy-five patients were carriers of a mutation in one of the FTD-associated genes: 27 with a mutation in *MAPT* [16], [17], 18 in *GRN* [18], [19], 29 in chromosome 9 open reading frame 72 (*C9orf72*) [20], [21], and one with a dual mutation in both *GRN* and *C9orf72*, who was excluded from the genetic analysis. For 79 patients, *postmortem* confirmation of the underlying neuropathology was available: fused in sarcoma (n = 3), TDP-43 type A (n = 16), TDP-43 type B (n = 3), TDP-43 type C (n = 20); Pick's disease (n = 17), progressive supranuclear palsy (n = 4), corticobasal degeneration (n = 9), and frontotemporal dementia with parkinsonism linked to chromosome 17 (FTDP-17) (n = 7). There was an overlap of 21 cases between the genetic groups and the pathological groups: 5 patients with *GRN* mutation had TDP-43 type A, 7 with *C9orf72* mutation had TDP-43 type A and 2 had TDP-43 type B, and 7 with *MAPT* mutation had FTDP-17; the patient with dual *GRN/C9orf72* mutation had TDP-43 type A. A total of 107 cognitively normal subjects, with a similar age to the patients and a usable volumetric T1-weighted MRI, were identified as controls. The study was approved by the local ethics committee, and written informed consent was obtained from all participants.

**Table 1:** Demographic and clinical characteristics of the FTD cohort

Groups	Subgroups	n	Clinical diagnosis (bvFTD/FTD-MND/svPPA/nfvPPA/PPA-NOS)	Age, years, mean (SD)	Gender (% of male)	Disease duration, years, mean (SD)	Scanner (1.5 T GE/3T Siemens Trio/3T Siemens Prisma)
Controls		107	—	62.7 (11.3)	44%	—	35/56/16
Genetic	*MAPT*	27	26/0/0/1/0	56.0 (7.6)	63%	5.7 (3.2)	13/11/3
	*GRN*	18	11/0/0/5/2	62.0 (6.4)	56%	3.2 (2.8)	8/5/5
	*C9orf72*	29	24/3/0/2/0	62.1 (6.8)	69%	5.5 (3.2)	10/14/5
Pathology	FTDP-17	7	7/0/0/0/0	51.3 (5.8)	71%	5.2 (3.1)	6/1/0
	Tau-Pick's	17	9/0/3/4/1	59.7 (4.2)	76%	4.4 (2.2)	14/3/0
	Tau-PSP	4	2/0/0/2/0	77.1 (7.6)	100%	5.4 (4.4)	2/1/1
	Tau-CBD	9	5/0/0/4/0	61.9 (9.2)	78%	4.6 (0.9)	6/3/0
	TDP-43 type A	16	11/1/0/3/1	60.9 (7.4)	63%	3.4 (1.7)	9/7/0
	TDP-43 type B	3	3/0/0/0/0	57.1 (7.7)	67%	4.8 (2.7)	3/0/0
	TDP-43 type C	20	0/0/19/1/0	65.3 (7.3)	65%	4.7 (2.7)	16/4/0
	FUS	3	3/0/0/0/0	43.9 (13.6)	67%	3.3 (2.1)	3/0/0

Abbreviations: bvFTD, behavioral variant of frontotemporal dementia; FTD-MND, frontotemporal dementia with associated motor neurone disease; svPPA, semantic variant of primary progressive aphasia; nfvPPA, nonfluent variant of primary progressive aphasia; PPA-NOS, primary progressive aphasia not otherwise specified; SD, standard deviation; FTDP-17, frontotemporal dementia with Parkinsonism linked to chromosome 17; PSP, progressive supranuclear palsy; CBD, corticobasal degeneration; TDP-43, transactive response DNA binding protein 43 kDa; FUS, fused in sarcoma.

No significant age difference was seen between the FTD groups and controls (Table 1). Among the genetic groups, the *GRN* group had a shorter disease duration (3.2 [2.8] years) than the *C9orf72* (5.5 [3.2], *P* = .040) and *MAPT* (5.7 [3.2], *P* = .002) groups, but no difference for disease duration was found among the pathological groups (*P* = .722, analysis of variance). No difference was found among controls and genetic groups for the type of scanner used (*P* = .413, chi-square test), but there was a difference for the pathological groups (*P* < .0005, chi-square test).

T1-weighted MRIs were acquired from 1993 to 2018 using scanners from three different manufacturers: 110 on 1.5 T Signa MRI scanner (GE Medical systems, Milwaukee, WI, repetition time = 12 ms, inversion time = 650 ms, echo time = 5 ms, acquisition matrix = 256 × 256, spatial resolution = 1.5 mm), 99 on 3T Trio MRI scanner (Siemens, Erlangen, Germany, repetition time = 2200 ms, inversion time = 900 ms, echo time = 2.9 ms, acquisition matrix = 256 × 256, spatial resolution = 1.1 mm), and 30 on 3T Prisma MRI scanner (Siemens, Erlangen, Germany, repetition time = 2000 ms, inversion time = 850 ms, echo time = 2.93 ms, acquisition matrix = 256 × 256, spatial resolution = 1.1 mm).

Volumetric MRI scans were first bias field corrected and whole-brain parcellated using the geodesic information flow (GIF) algorithm [22], which is based on atlas propagation and label fusion. Volumes of the whole amygdala and of 9 AS were subsequently segmented using a customized version of the module available in FreeSurfer 6.0 [15] to adapt the output of GIF to the FreeSurfer format. Based on anatomical subdivision [9], we combined the nine original subnuclei and focused the analysis on the following five regions: (1) lateral nucleus; (2) basal and paralaminar nucleus; (3) accessory basal nucleus (AB); (4) corticoamygdaloid transition area; and (5) the superficial nuclei (central nucleus, cortical nucleus, medial nucleus, anterior amygdaloid area) (Fig. 1). Based on the volumes of the left and right hemispheres extracted from the GIF, we defined the most severely affected cerebral hemisphere in each patient. This allows avoidance of the difficulties that commonly arise in imaging analyses of FTD in which accurate outcomes of volumetric analyses can be obfuscated by combining people with predominantly right- or left-hemisphere atrophy in the same analysis. We then compared AS volumes between groups within the most and least severely affected hemispheres rather than within right and left sides. However, we also investigated asymmetry by calculating an asymmetry index (AI), defined as the absolute difference between the left and right total amygdalar volumes in relation to the total bilateral volume: |(Left − Right)|/(Left + Right).

AS volumes were expressed as a percentage of the total intracranial volume, computed using SPM12 v6470 (Statistical Parametric Mapping; Wellcome Trust Center for Neuroimaging, London, UK) running under Matlab R2014b (Math Works, Natick, MA) [23]. All segmentations were visually checked for quality.

Statistical analyses were performed on AS volumes and AI using the SPSS software (SPSS Inc., Chicago, IL) v22.0, between control and FTD groups, using an analysis of variance test adjusting for the scanner type, total intracranial volume, gender, and age. Results were corrected for multiple comparisons (Bonferroni's correction) at *P* < .005 for the genetic and pathological groups.

## 3 Results

**Fig. 2:**
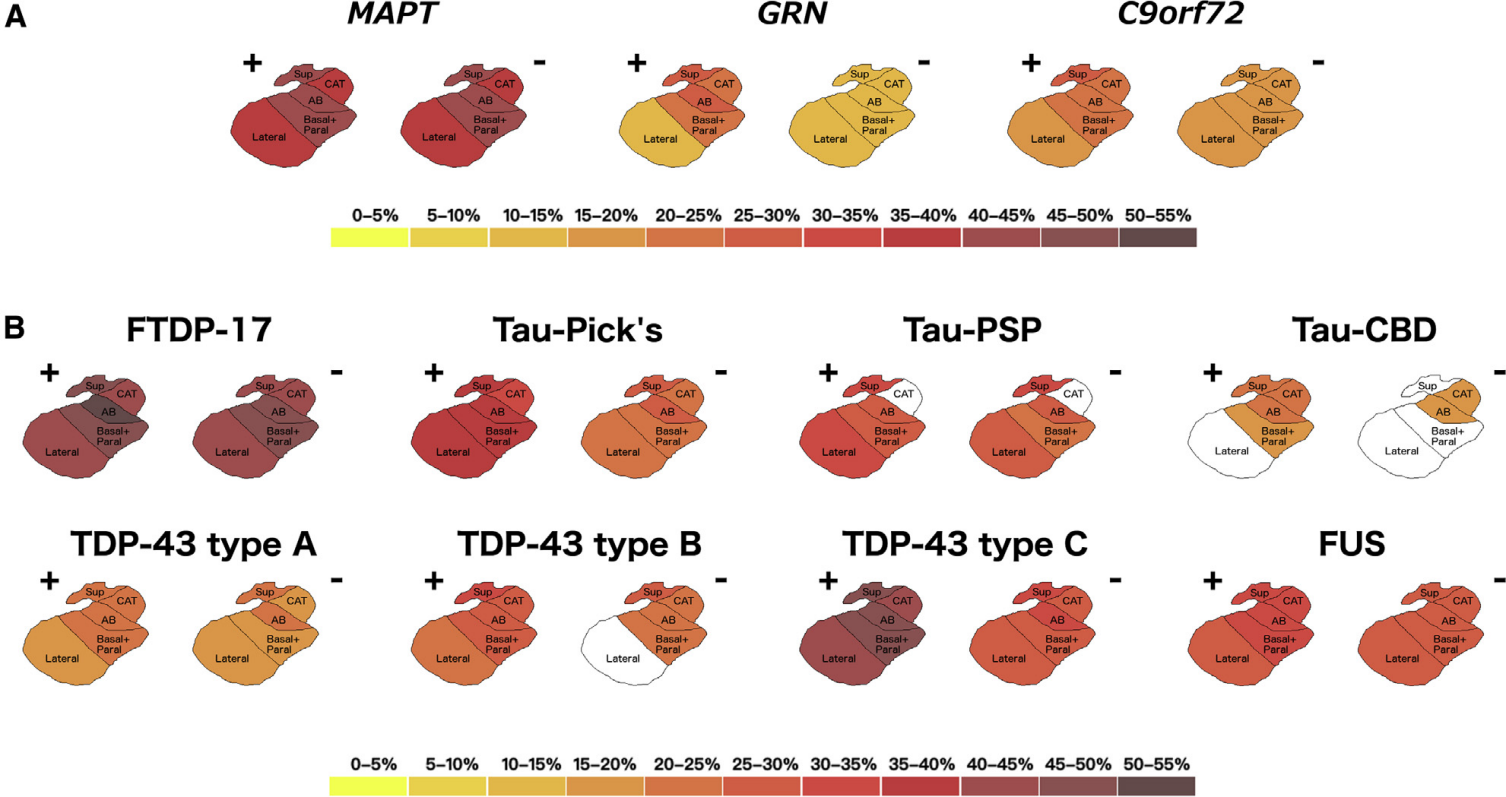
The pattern of atrophy in the amygdala subnuclei in the genetic (A) and pathologically confirmed (B) FTD cases. The color bar denotes the percentage difference in volume from controls. + denotes the most affected hemisphere; − denotes the least-affected hemisphere. FTD, frontotemporal dementia.

Stratifying by genetics, the *MAPT* group had the smallest AS in both hemispheres (37%–43%, *P* < .0005), whereas *C9orf72* and *GRN* had similar volume differences in the most affected hemisphere, with the greatest involvement in the superficial group and AB (24%–29%, *P* < .0005) and the lowest in the lateral nucleus (14%–18%, *P* < .0005) (Fig. 2A and Supplementary Table 1). All AS were significantly smaller in *MAPT* than in *C9orf72* and *GRN* (*P* < .0005; Supplementary Table 2).

Stratifying by pathology, the FTDP-17, TDP-43 type C, and Pick's disease groups were the most impaired, especially for the superficial (39%–47%), AB (38%–50%), and basal/paralaminar subnuclei (35%–39%, *P* < .0005) in the most affected hemisphere (Fig. 2B and Supplementary Table 1). Similar to *MAPT* mutations, the FTDP-17 group showed severe volumetric differences compared with controls in the least-affected hemisphere as well (AB and basal/paralaminar subnuclei: 47%–48%, *P* < .0005). In TDP-43 type A and type B, the smallest AS when compared with controls were the superficial and AB in both hemispheres (21%–24% and 25%–30% for superficial; 20%–23% and 23%–28% for AB, respectively, *P* < .003). Fused in sarcoma showed a homogenous involvement across all AS (28%–31% for the most affected side and 26%–28% for the least affected, *P* < .003). The most affected AS in progressive supranuclear palsy was of the superficial group (30%–33%), but without involvement of the corticoamygdaloid transition area. Corticobasal degeneration appeared to be the least-affected group (18%–24%, *P* < .0005) (Fig. 2B and Supplementary Table 1). Comparisons between the pathological groups are reported in the Supplementary Table 2.

For most groups, no differences were seen between the right and left amygdalae, except for the *GRN* (0.067 [0.060]), Pick's disease (0.110 [0.065]), corticobasal degeneration (0.075 [0.064]), and TDP-43 type C (0.140 [0.072]) groups, which had an AI significantly greater than that of controls (0.030 [0.026]) (*P* < .003).

## 4 Discussion

In a cohort of patients with genetic and pathologically confirmed FTD, we have shown that AS are the most affected in *MAPT*/FTDP-17, Pick's disease, and TDP-43 type C groups, with the most affected subnuclei across most groups being the superficial, AB, and basal/paralaminar ones.

Our results showing amygdalar involvement across all forms of FTD are consistent with and extend the work of previous imaging and neuropathological studies [7], [8], [24], [25]. Similarly, our work is consistent with prior studies showing particular involvement of the amygdala in patients with *MAPT* mutations/FTDP-17 pathology [4], [5], [6].

However, in this study, we were able to determine *in vivo* localization of the volume difference within the amygdala, where the most affected nuclei were the superficial, AB, and basal/paralaminar nuclei, which form part of the reward system, and these were also found to be connected to key limbic brain regions (Fig. 1). Impairment of the reward system is regularly seen in patients with FTD but is not universal to all people; previous studies have shown problems with changes in appetite and sexual behavior in patients with FTD due to *MAPT* mutations, Pick's disease, and TDP-43 type C [26], which were the most frequently diagnosed condition as reported in this study. AS are also involved in emotional processing, which is known to be impaired in both behavioral variant of frontotemporal dementia and semantic variant of primary progressive aphasia (which form the majority of the TDP-43 type C cases) [27]. Consistent with our findings, one previous functional magnetic resonance imaging study [28] has shown that specific AS associated with difficulty in recognizing facial emotional expressions in people with behavioral variant of frontotemporal dementia are the superficial and basolateral nuclei.

The reason for differential AS involvement in the different genetic and pathological forms of FTD and how this may lead to different symptoms is currently unclear. It is likely that different neuronal networks have specific vulnerability to particular pathological proteins, and our work here suggests that specific AS may be vulnerable in different proteinopathies [29] rather than the entire amygdala. Future research, brain imaging, both on a macroscopic level and at the cellular level, is needed to investigate this further.

Limitations of this study include the use of different scanners (three manufacturers, two different magnetic fields: 1.5T and 3T) with slightly different MRI sequence types. Even if we correct for the scanner type and gender in the statistical model, we cannot completely remove some of the intrinsic heterogeneity due to these variables. We used an automated method to extract the AS volumes, which is not as accurate as their manual segmentation on dedicated MRIs or on brain tissue *postmortem*, but we combined the smallest AS into larger groups of nuclei to remove the effect of their less reliable delineation on T1 MRIs and manual segmentation is extremely time-consuming and labor-intensive in such a large cohort.

Future studies of functional and diffusion MRI will be helpful to investigate, in detail, the different connections of the AS in each form of FTD. Furthermore, longitudinal studies, potentially including mutation carriers in their presymptomatic stage, will help to understand the differential involvement of AS over the course of the disease.

**Research in Context**

1. Systematic review: The amygdala is known to be atrophic in cases of frontotemporal dementia (FTD), but no prior study has specifically investigated the different amygdalar subnuclei. We reviewed the existing literature on PubMed Central on imaging and pathological studies of the amygdala in cases with FTD.
2. Interpretation: We showed *in vivo* differential amygdalar involvement across all forms of FTD. Our findings are consistent with and extend the previous studies. We showed that the most affected subnuclei form part of a network of regions that control reward and emotion regulation, functions known to be particularly affected in FTD.
3. Future directions: Further studies on connectivity of the amygdalar subnuclei, together with longitudinal studies, including presymptomatic mutation carriers, are key to better understand the differential involvement of the amygdalar subnuclei over the course of the disease.
