# Supplementary material for: Amygdala subnuclei are differentially affected in the different genetic and pathological forms of frontotemporal dementia
Source: Alzheimers Dement (Amst). 2019 Jan 25;11:136–41. doi: 10.1016/j.dadm.2018.12.006 (PMC6369146; doi:10.1016/j.dadm.2018.12.006)
Supplement: Supplementary Tables 1 and 2 [file mmc1.docx]

**Supplementary Table 1.** **Volumetric comparisons of the amygdalar subnuclei between the different genetic and pathological FTD subgroups and the controls for the most and least affected hemispheres.** Volumetric comparisons are adjusted for age, gender and scanner type. Bold represents a significant difference between groups after correcting for multiple comparisons.

|  | **Most Affected Hemisphere** | | | | | **Least Affected Hemisphere** | | | | |
| --- | --- | --- | --- | --- | --- | --- | --- | --- | --- | --- |
| **Genetic** | **Superficial** | **CAT** | **AB** | **Basal-Paralaminar** | **Lateral** | **Superficial** | **CAT** | **AB** | **Basal-Paralaminar** | **Lateral** |
| **control** | 0.011 (0.002) | 0.012 (0.002) | 0.018 (0.002) | 0.033 (0.004) | 0.046 (0.004) | 0.011 (0.002) | 0.012 (0.002) | 0.018 (0.002) | 0.033 (0.004) | 0.047 (0.005) |
| ***MAPT*** | 0.007 (0.002) | 0.008 (0.002) | 0.010 (0.002) | 0.019 (0.004) | 0.028 (0.005) | 0.007 (0.002) | 0.007 (0.002) | 0.010 (0.002) | 0.019 (0.005) | 0.029 (0.005) |
| ***GRN*** | 0.008 (0.002) | 0.009 (0.002) | 0.013 (0.003) | 0.025 (0.005) | 0.039 (0.006) | 0.010 (0.002) | 0.011 (0.002) | 0.015 (0.003) | 0.029 (0.005) | 0.043 (0.006) |
| ***C9orf72*** | 0.008 (0.001) | 0.010 (0.002) | 0.013 (0.002) | 0.026 (0.004) | 0.038 (0.006) | 0.009 (0.002) | 0.010 (0.002) | 0.014 (0.003) | 0.027 (0.004) | 0.039 (0.006) |
|  | | | | | | | | | | |
| **Control vs *MAPT*** | **41%** | **37%** | **43%** | **43%** | **39%** | **40%** | **39%** | **43%** | **41%** | **39%** |
|  | **<0.0005** | **<0.0005** | **<0.0005** | **<0.0005** | **<0.0005** | **<0.0005** | **<0.0005** | **<0.0005** | **<0.0005** | **<0.0005** |
| **Control vs *GRN*** | **29%** | **24%** | **25%** | **23%** | **14%** | **14%** | **12%** | **13%** | **11%** | **10%** |
|  | **<0.0005** | **<0.0005** | **<0.0005** | **<0.0005** | **<0.0005** | **0.001** | **0.001** | **<0.0005** | **<0.0005** | **0.001** |
| **Control vs *C9orf72*** | **25%** | **20%** | **24%** | **21%** | **18%** | **19%** | **18%** | **19%** | **17%** | **18%** |
|  | **<0.0005** | **<0.0005** | **<0.0005** | **<0.0005** | **<0.0005** | **<0.0005** | **<0.0005** | **<0.0005** | **<0.0005** | **<0.0005** |
|  | | | | | | | | | | |
|  | **Most Affected Hemisphere** | | | | | **Least Affected Hemisphere** | | | | |
| **Pathology** | **Superficial** | **CAT** | **AB** | **Basal-Paralaminar** | **Lateral** | **Superficial** | **CAT** | **AB** | **Basal-Paralaminar** | **Lateral** |
| **control** | 0.011 (0.002) | 0.012 (0.002) | 0.018 (0.002) | 0.033 (0.004) | 0.046 (0.004) | 0.011 (0.002) | 0.012 (0.002) | 0.018 (0.002) | 0.033 (0.004) | 0.047 (0.005) |
| **FTDP-17** | 0.006 (0.001) | 0.007 (0.001) | 0.009 (0.001) | 0.017 (0.002) | 0.026 (0.003) | 0.006 (0.001) | 0.007 (0.001) | 0.009 (0.001) | 0.017 (0.002) | 0.028 (0.005) |
| **Tau-Pick's** | 0.007 (0.002) | 0.008 (0.002) | 0.011 (0.002) | 0.021 (0.004) | 0.029 (0.007) | 0.008 (0.001) | 0.009 (0.001) | 0.013 (0.002) | 0.026 (0.004) | 0.037 (0.006) |
| **Tau-PSP** | 0.007 (0.002) | 0.009 (0.002) | 0.013 (0.002) | 0.023 (0.005) | 0.032 (0.005) | 0.008 (0.002) | 0.009 (0.002) | 0.013 (0.003) | 0.025 (0.006) | 0.034 (0.007) |
| **Tau-CBD** | 0.009 (0.002) | 0.009 (0.002) | 0.014 (0.003) | 0.027 (0.006) | 0.040 (0.007) | 0.009 (0.002) | 0.010 (0.002) | 0.015 (0.002) | 0.029 (0.004) | 0.042 (0.005) |
| **TDP-43 Type A** | 0.009 (0.001) | 0.009 (0.001) | 0.014 (0.002) | 0.026 (0.003) | 0.037 (0.005) | 0.009 (0.001) | 0.010 (0.001) | 0.014 (0.002) | 0.027 (0.003) | 0.038 (0.004) |
| **TDP-43 Type B** | 0.008 (0.002) | 0.009 (0.001) | 0.013 (0.003) | 0.025 (0.005) | 0.037 (0.010) | 0.008 (0.002) | 0.010 (0.002) | 0.014 (0.003) | 0.026 (0.007) | 0.040 (0.010) |
| **TDP-43 Type C** | 0.006 (0.001) | 0.007 (0.001) | 0.009 (0.001) | 0.017 (0.002) | 0.025 (0.004) | 0.008 (0.001) | 0.009 (0.002) | 0.012 (0.002) | 0.024 (0.004) | 0.034 (0.006) |
| **FUS** | 0.008 (0.002) | 0.008 (0.002) | 0.012 (0.002) | 0.023 (0.004) | 0.033 (0.005) | 0.008 (0.001) | 0.009 (0.001) | 0.013 (0.001) | 0.024 (0.003) | 0.034 (0.005) |
|  | | | | | | | | | | |
| **Control vs FTDP-17** | **47%** | **43%** | **50%** | **49%** | **42%** | **42%** | **44%** | **47%** | **48%** | **40%** |
|  | **<0.0005** | **<0.0005** | **<0.0005** | **<0.0005** | **<0.0005** | **<0.0005** | **<0.0005** | **<0.0005** | **<0.0005** | **<0.0005** |
| **Control vs Tau-Pick's** | **39%** | **32%** | **38%** | **35%** | **37%** | **27%** | **23%** | **26%** | **22%** | **22%** |
|  | **<0.0005** | **<0.0005** | **<0.0005** | **<0.0005** | **<0.0005** | **<0.0005** | **<0.0005** | **<0.0005** | **<0.0005** | **<0.0005** |
| **Control vs Tau-PSP** | **33%** | 23% | **28%** | **29%** | **31%** | **30%** | 26% | **25%** | **24%** | **27%** |
|  | **0.001** | 0.03 | **0.001** | **<0.0005** | **<0.0005** | **0.003** | 0.005 | **0.001** | **0.001** | **<0.0005** |
| **Control vs Tau-CBD** | **24%** | **21%** | **22%** | **19%** | 13% | 18% | **18%** | **18%** | 13% | 10% |
|  | **<0.0005** | **<0.0005** | **<0.0005** | **<0.0005** | 0.006 | 0.01 | **0.002** | **0.001** | 0.016 | 0.04 |
| **Control vs TDP-43 Type A** | **24%** | **20%** | **23%** | **21%** | **18%** | **21%** | **18%** | **20%** | **17%** | **19%** |
|  | **<0.0005** | **<0.0005** | **<0.0005** | **<0.0005** | **<0.0005** | **<0.0005** | **<0.0005** | **<0.0005** | **<0.0005** | **<0.0005** |
| **Control vs TDP-43 Type B** | **30%** | **21%** | **28%** | **25%** | **20%** | **25%** | **20%** | **23%** | **20%** | 16% |
|  | **<0.0005** | **0.004** | **<0.0005** | **<0.0005** | **0.001** | **0.003** | **0.004** | **0.002** | **0.004** | 0.011 |
| **Control vs TDP-43 Type C** | **45%** | **43%** | **48%** | **48%** | **44%** | **32%** | **28%** | **30%** | **28%** | **29%** |
|  | **<0.0005** | **<0.0005** | **<0.0005** | **<0.0005** | **<0.0005** | **<0.0005** | **<0.0005** | **<0.0005** | **<0.0005** | **<0.0005** |
| **Control vs FUS** | **30%** | **31%** | **30%** | **31%** | **28%** | **26%** | **28%** | **28%** | **26%** | **27%** |
|  | **<0.0005** | **<0.0005** | **<0.0005** | **<0.0005** | **<0.0005** | **0.003** | **<0.0005** | **<0.0005** | **<0.0005** | **<0.0005** |

**Supplementary Table 2.** **Volumetric comparisons of the amygdalar subnuclei between the different genetic and pathological FTD subgroups or the most and least affected hemispheres.** Volumetric comparisons are adjusted for age, gender and scanner type. Bold represents a significant difference between groups after correcting for multiple comparisons.

|  |  | **Most Affected Hemisphere** | | | | | | | | | | **Least Affected Hemisphere** | | | | | | | | | |
| --- | --- | --- | --- | --- | --- | --- | --- | --- | --- | --- | --- | --- | --- | --- | --- | --- | --- | --- | --- | --- | --- |
|  |  | **Superficial** | | **CAT** | | **AB** | | **Basal-Paralaminar** | | **Lateral** | | **Superficial** | | **CAT** | | **AB** | | **Basal-Paralaminar** | | **Lateral** | |
|  |  | **%** | **P** | **%** | **P** | **%** | **P** | **%** | **P** | **%** | **P** | **%** | **P** | **%** | **P** | **%** | **P** | **%** | **P** | **%** | **P** |
| ***MAPT*** | ***GRN*** | **-22** | **<0.0005** | **-20** | **<0.0005** | **-32** | **<0.0005** | **-35** | **<0.0005** | **-41** | **<0.0005** | **-43** | **<0.0005** | **-45** | **<0.0005** | **-54** | **<0.0005** | **-51** | **<0.0005** | **-47** | **<0.0005** |
|  | ***C9orf72*** | **-28** | **<0.0005** | **-27** | **<0.0005** | **-34** | **<0.0005** | **-38** | **<0.0005** | **-36** | **<0.0005** | **-34** | **<0.0005** | **-35** | **<0.0005** | **-42** | **<0.0005** | **-41** | **<0.0005** | **-34** | **<0.0005** |
| ***GRN*** | ***C9orf72*** | -5 | 0.5 | -5 | 0.464 | -1 | 0.972 | -2 | 0.707 | 4 | 0.183 | 6 | 0.131 | 7 | 0.079 | 8 | 0.044 | 6 | 0.088 | **9** | **0.004** |
|  |  | **Most Affected Hemisphere** | | | | | | | | | | **Least Affected Hemisphere** | | | | | | | | | |
|  |  | **Superficial** | | **CAT** | | **AB** | | **Basal-Paralaminar** | | **Lateral** | | **Superficial** | | **CAT** | | **AB** | | **Basal-Paralaminar** | | **Lateral** | |
|  |  | **%** | **P** | **%** | **P** | **%** | **P** | **%** | **P** | **%** | **P** | **%** | **P** | **%** | **P** | **%** | **P** | **%** | **P** | **%** | **P** |
| **FTDP-17** | **Tau-Pick's** | -15 | 0.118 | -18 | 0.036 | -23 | 0.016 | **-28** | **0.003** | -10 | 0.185 | -27 | 0.011 | **-37** | **<0.0005** | **-38** | **<0.0005** | **-49** | **<0.0005** | **-31** | **<0.0005** |
|  | **Tau-PSP** | -26 | 0.012 | **-34** | **<0.0005** | **-43** | **<0.0005** | **-40** | **0.001** | -20 | 0.009 | -22 | 0.03 | **-31** | **0.002** | **-41** | **0.001** | **-44** | **<0.0005** | -22 | 0.011 |
|  | **Tau-CBD** | **-43** | **<0.0005** | **-37** | **<0.0005** | **-56** | **<0.0005** | **-59** | **<0.0005** | **-51** | **<0.0005** | **-43** | **<0.0005** | **-47** | **<0.0005** | **-55** | **<0.0005** | **-66** | **<0.0005** | **-51** | **<0.0005** |
|  | **TDP-43 Type A** | **-42** | **<0.0005** | **-39** | **<0.0005** | **-53** | **<0.0005** | **-55** | **<0.0005** | **-42** | **<0.0005** | **-37** | **0.001** | **-45** | **<0.0005** | **-51** | **<0.0005** | **-58** | **<0.0005** | **-36** | **<0.0005** |
|  | **TDP-43 Type B** | -31 | 0.068 | -38 | 0.011 | -43 | 0.006 | **-48** | **0.001** | **-39** | **0.003** | -30 | 0.068 | -42 | 0.008 | **-45** | **0.003** | **-53** | **<0.0005** | **-41** | **0.001** |
|  | **TDP-43 Type C** | -3 | 0.343 | 1 | 0.437 | -4 | 0.238 | -3 | 0.383 | 3 | 0.922 | -19 | 0.022 | **-29** | **<0.0005** | **-31** | **<0.0005** | **-38** | **<0.0005** | **-19** | **0.004** |
|  | **FUS** | -32 | 0.108 | -21 | 0.253 | -39 | 0.025 | -36 | 0.024 | -25 | 0.058 | -29 | 0.087 | -28 | 0.096 | -35 | 0.021 | -42 | 0.006 | -22 | 0.086 |
| **Tau-Pick's** | **Tau-PSP** | -9 | 0.096 | -14 | 0.016 | -16 | 0.02 | -10 | 0.096 | -10 | 0.05 | 4 | 0.637 | 5 | 0.557 | -2 | 0.396 | 3 | 0.772 | 7 | 0.87 |
|  | **Tau-CBD** | **-24** | **0.002** | -16 | 0.007 | **-27** | **<0.0005** | **-25** | **<0.0005** | **-37** | **<0.0005** | -13 | 0.024 | -7 | 0.1 | -12 | 0.019 | -11 | 0.017 | **-15** | **0.002** |
|  | **TDP-43 Type A** | **-24** | **0.003** | -18 | 0.005 | **-24** | **<0.0005** | **-21** | **0.001** | **-29** | **<0.0005** | -8 | 0.207 | -6 | 0.253 | -9 | 0.09 | -6 | 0.247 | -4 | 0.323 |
|  | **TDP-43 Type B** | -14 | 0.375 | -17 | 0.197 | -16 | 0.192 | -16 | 0.166 | -26 | 0.017 | -3 | 0.879 | -3 | 0.872 | -5 | 0.653 | -2 | 0.861 | -8 | 0.376 |
|  | **TDP-43 Type C** | 11 | 0.395 | 16 | 0.07 | 15 | 0.09 | **19** | **0.004** | 12 | 0.095 | 6 | 0.696 | 6 | 0.666 | 5 | 0.655 | 8 | 0.268 | 8 | 0.154 |
|  | **FUS** | -15 | 0.531 | -2 | 0.792 | -13 | 0.472 | -7 | 0.757 | -14 | 0.266 | -1 | 0.981 | 6 | 0.356 | 3 | 0.736 | 5 | 0.459 | 7 | 0.349 |
| **Tau-PSP** | **Tau-CBD** | -14 | 0.589 | -2 | 0.662 | -9 | 0.651 | -14 | 0.293 | -25 | 0.028 | -17 | 0.269 | -12 | 0.569 | -10 | 0.416 | -15 | 0.175 | -24 | 0.02 |
|  | **TDP-43 Type A** | -13 | 0.896 | -4 | 0.467 | -7 | 0.883 | -11 | 0.651 | -18 | 0.26 | -12 | 0.768 | -11 | 0.916 | -8 | 0.855 | -9 | 0.679 | -12 | 0.443 |
|  | **TDP-43 Type B** | -5 | 0.6 | -3 | 0.449 | 0 | 0.497 | -6 | 0.899 | -15 | 0.64 | -6 | 0.821 | -8 | 0.761 | -3 | 0.788 | -6 | 0.941 | -16 | 0.411 |
|  | **TDP-43 Type C** | 18 | 0.027 | **26** | **<0.0005** | **27** | **0.001** | **26** | **0.001** | **20** | **0.003** | 3 | 0.472 | 2 | 0.388 | 7 | 0.254 | 4 | 0.338 | 2 | 0.495 |
|  | **FUS** | -5 | 0.484 | 10 | 0.053 | 3 | 0.271 | 3 | 0.342 | -4 | 0.596 | -5 | 0.75 | 2 | 0.252 | 4 | 0.383 | 2 | 0.429 | 0 | 0.533 |
| **Tau-CBD** | **TDP-43 Type A** | 1 | 0.541 | -1 | 0.718 | 2 | 0.388 | 3 | 0.358 | 6 | 0.094 | 4 | 0.225 | 1 | 0.492 | 2 | 0.347 | 5 | 0.156 | 9 | 0.019 |
|  | **TDP-43 Type B** | 8 | 0.268 | 0 | 0.625 | 8 | 0.228 | 7 | 0.268 | 8 | 0.144 | 9 | 0.203 | 3 | 0.381 | 6 | 0.291 | 8 | 0.185 | 6 | 0.244 |
|  | **TDP-43 Type C** | **28** | **<0.0005** | **28** | **<0.0005** | **33** | **<0.0005** | **35** | **<0.0005** | **36** | **<0.0005** | 17 | 0.008 | 12 | 0.04 | 15 | 0.005 | **17** | **0.001** | **21** | **<0.0005** |
|  | **FUS** | 8 | 0.187 | 12 | 0.056 | 11 | 0.087 | 15 | 0.039 | 17 | 0.009 | 10 | 0.17 | 12 | 0.061 | 13 | 0.077 | 15 | 0.031 | 19 | 0.005 |
| **TDP-43 Type A** | **TDP-43 Type B** | 8 | 0.439 | 1 | 0.779 | 6 | 0.475 | 5 | 0.569 | 2 | 0.657 | 5 | 0.582 | 3 | 0.634 | 4 | 0.616 | 3 | 0.639 | -4 | 0.743 |
|  | **TDP-43 Type C** | **28** | **<0.0005** | **29** | **<0.0005** | **32** | **<0.0005** | **34** | **<0.0005** | **32** | **<0.0005** | 14 | 0.09 | 11 | 0.107 | 13 | 0.028 | 13 | 0.022 | 12 | 0.015 |
|  | **FUS** | 7 | 0.319 | 13 | 0.075 | 9 | 0.211 | 12 | 0.111 | 12 | 0.096 | 6 | 0.508 | 12 | 0.126 | 11 | 0.209 | 10 | 0.174 | 11 | 0.144 |
| **TDP-43 Type B** | **TDP-43 Type C** | 22 | 0.179 | 28 | 0.024 | 27 | 0.027 | **30** | **0.003** | **30** | **0.001** | 9 | 0.717 | 9 | 0.694 | 10 | 0.49 | 10 | 0.441 | 15 | 0.099 |
|  | **FUS** | 0 | 0.851 | 12 | 0.236 | 3 | 0.664 | 8 | 0.415 | 9 | 0.332 | 1 | 0.923 | 9 | 0.402 | 7 | 0.547 | 7 | 0.479 | 14 | 0.162 |
| **TDP-43 Type C** | **FUS** | -29 | 0.286 | -22 | 0.493 | -33 | 0.11 | -32 | 0.069 | -30 | 0.048 | -8 | 0.82 | 0 | 0.486 | -3 | 0.918 | -3 | 0.872 | -2 | 0.849 |
